# The role of immune cells-mediated memory in weight cycling, glucose disorders and insulin resistance

**DOI:** 10.3389/fimmu.2025.1721553

**Published:** 2025-12-18

**Authors:** Yiding Chen, Dongqi Zhou, Lan Wang, Lisha Sun, Yun Yin, Guo Liu, Changyan Zi

**Affiliations:** 1 Qionglai Hospital of Traditional Chinese Medicine, Chengdu, Sichuan, China; 2 Hospital of Chengdu University of Traditional Chinese Medicine, Chengdu, China; 3 Sichuan Taikang Hospital, Tianfu New Area, Sichuan, China

**Keywords:** immune memory, weight cycling, macrophages, T-cells, insulin resistance

## Abstract

Weight cycling (WC), defined as the repeated process of weight gain and loss, is one of the biggest challenges in the management of weight. It is estimated that the majority of individuals (60%) will regain their lost weight within a few years. There is a positive correlation between WC and the increased risk of metabolic diseases. Although multiple factors probably contribute to this variation, immune cells-mediated immune memory plays a key role. In this review, we showed that immune memory is one of the core mechanisms of WC, glucose and insulin disorders. Immune cells, such as macrophages, CD4+ T cells, CD8+ T cells, Treg cells and CD7+ monocytes, were included. We also exhibited potential therapies to prevent WC targeting immune memory.

## 1 Introduction

Based on the NCD Risk Factor Collaboration (NCD-RisC) published findings in 2024, it is estimated that more than one billion people in the world were living with obesity, nearly 880 million adults (1). Although weight loss, known to improve metabolic outcomes associated with obesity, is highly recommended for those with obesity, recent studies have reported that most individuals (60%) will regain their lost weight within a few years (2, 3). One-third to two-thirds of the weight lost is regained within 1 year and almost all is regained within 5 years (4). Importantly, weight cycling (WC), the repeated process of weight gain and loss, is closely related to elevated risk for developing diabetes, compared to obese individuals who have never lost weight (5, 6).

**Figure 1:**
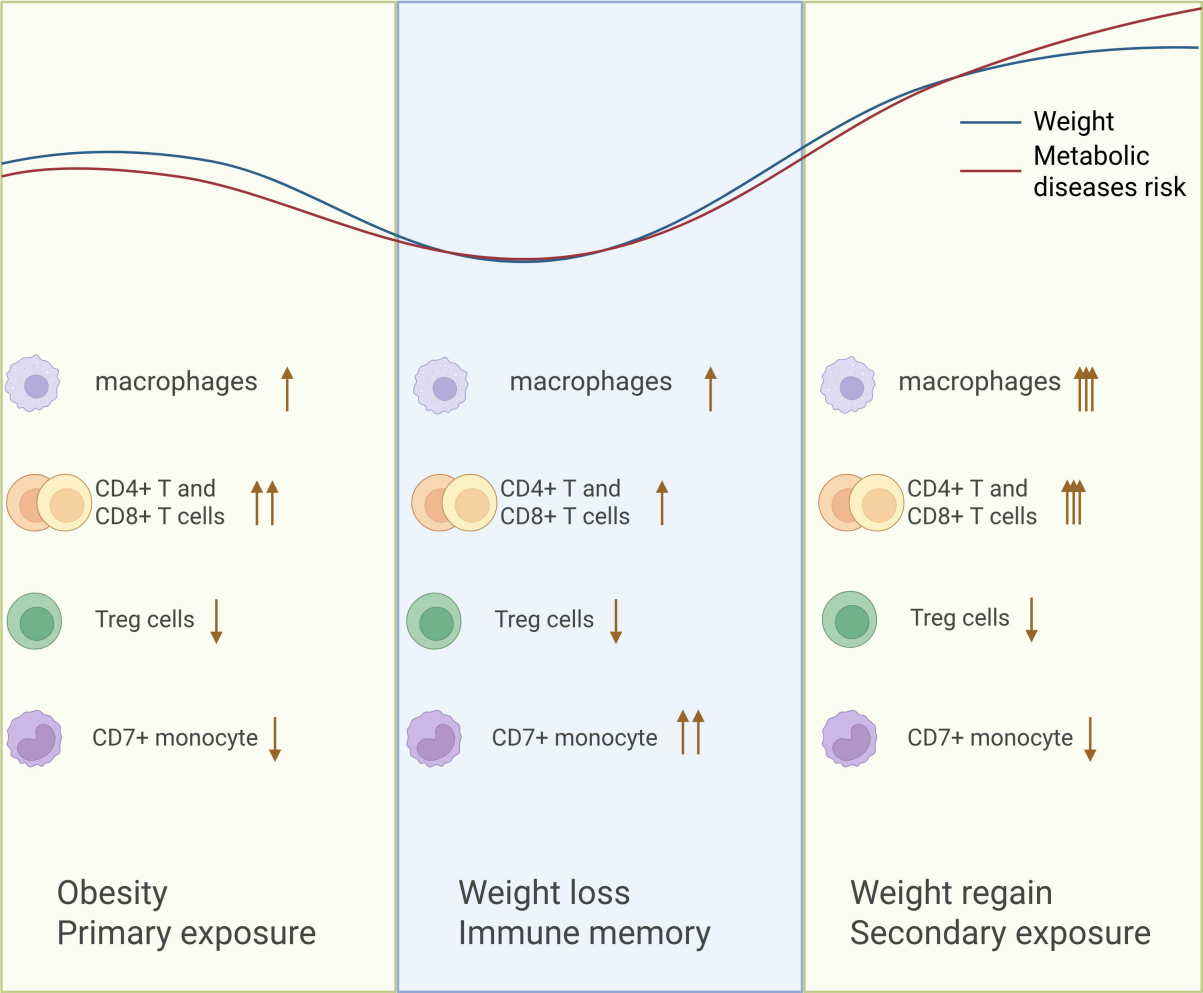
Graphic abstract. Initial metabolic stimuli induce pro-inflammatory memory in immune cells when obesity, which is activated during weight cycling and then aggravates metabolic diseases through enhanced inflammation and damaged metabolic homeostasis.

As low success rates of weight loss and failure to maintain weight are common, knowledge about the mechanisms resulting from WC is needed. A growing body of research has probed into the underlying mechanisms of WC over the past few years, including immune memory, gut microbiome, percentage of lost fat-free mass, appetite control, hormonal and neuronal factors, extracellular matrix remodeling and adaptive thermogenesis (7–9). However, despite extensive investigation, the obese imprint formed by immune memory, especially immune cells-mediated memory, is only partly understood. As we know, obesity is a state of chronic, low-grade inflammation, and adipose immune cell infiltration is the core source of chronic inflammation in adipose tissue. The present work aims to mechanistically integrate the role of immune cells-mediated memory in WC. Macrophages, CD4+ T and CD8+ T cells, Treg cells and CD7+ monocytes are involved (**Figure 1**).

## 2 Mechanisms of immune memory-mediated WC

Immune memory is a long-term adaptive modification of immune cells in response to stimuli, which is characterized by rapid activation when exposed to similar stimuli again (10). Adaptive memory is established by memory T and B lymphocytes following the recognition of an antigen (11), and innate immune memory, also called trained immunity, is made up of innate cells, such as macrophages and natural killer cells, through epigenetic and metabolic reprogramming (12, 13). Metabolic immune memory, in which immune cells are adaptively modified to metabolic stimuli such as obesity and high-fat diets, remains following weight loss and can trigger weight regain (14).

Adipose tissue expansion brings about a complicated and extensive immune response in human obesity, including the innate and adaptive immune system, which play essential roles in the modulation of AT inflammation (15). AT inflammation is largely caused or exacerbated by the following factors, such as immune cell recruitment rapidly, remodeling of the AT stromal immune components (e.g., immune cells, endothelial cells, fibroblasts and adipocyte progenitors), and AT immune cell dysfunction (16). Immune memory is formed due to chronic inflammatory stimulation, and is not reversed with weight loss. It has been believed that this kind of immune memory is the hallmark of innate and adaptive immune cells (17). Experiments have found that weight loss limited obesity-induced immune cells infiltration but did not totally reverse activation. These cells, like adipose tissue macrophages and memory T-cell subsets, were primed to potentially trigger weight regain and exacerbate metabolic dysfunction following subsequent stimulation (14, 18, 19). Remarkably, it was observed significant metabolic changes in a model of obese C57BL/6J mice fed with a high-fat diet and then switched to a low-calorie diet, resulting in a decrease of body weight to that of lean mice (20). Inflammation of the liver and perigonadal fat, persistently existed in formerly obese mice, revealing an upregulation in pathways related to immune function (Vcam1, Lyz1, H2-q5) and cellularity. Thus, sustained proinflammatory adipose tissue could result in an elevated risk of formerly obese subjects to develop the metabolic syndrome upon WC (20, 21). Following weight loss after bariatric surgery in humans, adipose tissue retains inflammation level as if it memorizes the obese state (22). It is established that obesogenic memory exists in mouse adipocytes and probably other cell types, largely mediated by stable epigenetic alterations (23).

## 3 Glucose disorders and insulin resistance following WC

The frequency and degree of WC are positively correlated with the increased risk of type 2 diabetes (24). A systematic review and meta-analysis involving 253,766 participants included 8,904 diabetes events, which showed that WC was a strong independent predictor of new-onset diabetes (25). WC enhances the risk of diabetes through impairing the function of β cells, β-cell endoplasmic reticulum stress and modulating the expression of insulin-secreting genes (26).

Weight loss induced a significant decrease in blood glucose and weight regain increased the blood glucose level back to the obese state (27). The findings from studies of experimentally induced WC have revealed that fluctuations of blood glucose and insulin levels repeatedly exceed normal values during periods of weight regain. This can put additional pressure on the metabolic system (4). In comparison with animals with later onset obesity, weight-cycled animals had higher fasting glucose levels and more impaired glucose tolerance following weight regain (28).

WC may also promote insulin resistance. Combat sports athletes often engage in weight management. Their rapid weight loss may decrease metabolic rate, insulin, and leptin. However, after competition, combat athletes may gain more weight than their original baseline. This kind of WC may be accompanied by signs of insulin resistance (29). A study about Japanese subjects has demonstrated that WC increased the risk of developing fasting hyperinsulinemia (30). In this cross-sectional analysis, weight histories of 1932 male Japanese workers aged 40–59 years were analyzed, and the researchers found a positive association between fasting insulin concentration and a history of weight fluctuations in the preceding 30 years. Moreover, individuals with larger weight fluctuations had significantly higher fasting insulin. Another study of WC in young German men, fasting and postprandial insulin sensitivity were also found to be impaired (31).

## 4 Immune cell-mediated memory

### 4.1 Macrophage - inflammation amplifier

Innate immune cells are primed by stimuli to enhance subsequent activation to a second stimulus. This response has been called ‘innate immune memory’ or ‘trained innate immunity’, which was initially found in β-glucan and the Mycobacterium tuberculosis vaccine (BCG), but has also been observed with cytokines, hormones, and oxidized low-density lipoprotein (32–34).

In adipose tissue, immune cells and adipose cells have a close interaction, shaping a complex microenvironment. Despite weight loss, adipose tissue inflammation persisted with elevated macrophage infiltration, pro-inflammatory markers and impaired Glut4 expression (35). The metabolic outcomes in mice with WC were worse, as shown by higher circulating IL-6 and leptin levels, increased hepatic lipid storage, and dysregulated glucose metabolism (35). Research related to selective remodeling of the adipose niche has demonstrated that extensive immune cells (mainly macrophage but also lymphocyte) infiltrate in obese AT (19). The adipose macrophages which express lysosomal, lipid metabolism and metabolic activation markers (CD9, TREM2, LPL and LIPA), would be remodeled in weight loss (19). Persistent macrophage activation that is probably epigenetically programmed in human AT may inhibit full metabolic recovery, resulting in WC and exacerbate long-term clinical outcomes (9, 19). Multiomics reveals persistence of obesity-associated immune cell phenotypes in adipose tissue during WC (36). Potential subpopulations of interest were identified by high-resolution subclusters, such as tissue resident macrophages (TRMs) abundant in the lean state and lipid-associated macrophages (LAMs) abundant in the obese state. TRMs decreased with obesity and even lower with weight loss. Notably, while LAMs increased with obesity, they did not return to lean levels with weight loss and increased even more with WC (36), which may be due to the fact that macrophages can remember the obesity status of the body.

Adipose macrophages, following weight loss, were primed for greater activation to subsequent stimulation by LPS ex vivo (14). During the WC, adipose macrophages have increased metabolism and released higher levels of basal TNF-α, suggesting that weight loss can prime macrophages for enhanced inflammation when weight is regained (14). Thus, this innate immune memory response exerts the deterioration of glucose tolerance following WC (14). Evidence of early alterations in adipose tissue of obese children already exhibited macrophage infiltration in adipose tissue and elevated circulating inflammatory markers (37). Immune cells (macrophage and T cell)-mediated inflammation in adipose tissue may contribute to the elevated type 2 diabetes risk in adults with childhood-onset obesity compared to those with adult-onset obesity (38).

### 4.2 CD4+ T and CD8+ T cells – long-term maintainers of metabolic abnormality

It was reported that obesity-associated inflammation changed T cell exhaustion, antigen presentation and lipid handling (36). During weight loss, adipose tissue inflammation was limited; however, memory T cells may be maintained within the tissue. Once subsequent weight regain and re-exposure to antigens in obese adipose tissue could result in a more potent and rapid memory T cell-mediated secondary immune response (39). In male mice, inflammation that remained following weight loss and deteriorated with weight regain suggested a memory-like immunological imprinting that may bring about WC-induced metabolic disease (36). Adaptive T cells are key regulators and drivers of inflammation in AT. WC increased activated T-cell accumulation in adipose tissue and promoted accumulation of TG in the muscle and liver, which further impairs systemic glucose tolerance and insulin sensitivity (19, 40). CD4+ T and CD8+ T cells were abundant in obese AT, remodeling effects ameliorated by weight loss (19). CD4+ T cells mediate obesity memory and promote weight regain. In a WC model, it was observed that activated CD4+ T cells were enhanced in C57BL/6J mice experiencing 1-month high-fat diet followed by weight normalization. In these mice, the rate of WC and adipose T-cell abundance increased compared to non-cycling mice (41). Whereas these differences disappeared with administration of dexamethasone, a T-cell and proinflammatory cytokine inhibitor (41). These results demonstrate that adaptive immune cells are the key condition for obesity memory development.

It was found that although the metabolic profile was ameliorated by weight loss, macrophage-mediated CD8+ T cell inflammation is actively ongoing in the liver and adipose tissue (42). Obese adults easily tend to regain weight even if they lose weight successfully (42). WC contributed to an additional increase in fasting blood glucose levels and glucose tolerance when compared with the obese mice (43). Additionally, WC resulted in a complete loss of insulin-stimulated AKT phosphorylation, indicating that WC further impaired AT insulin sensitivity. The potential mechanism may be that WC leads to an even further elevated percentage of both CD4+ and CD8+ T cells in AT (43). Some studies, however, hold different opinions about CD8+ T cells. The expression of exhaustion-associated genes in CD8+ T cells that is not normalized by weight loss is heightened by obesity (36). T cell exhaustion has been recently noted in human and mouse adipose tissue CD8+ T cells, and increased markers of T cell exhaustion were observed in obesity (44, 45). As T cell exhaustion would significantly inhibit the antigen-specific and or memory responses (44), a concept that is supported by the findings above that WC have susceptibility to CD8+ T cells-deficient condition. Another T cells also contribute to WC. A striking 4.5-fold increase in the expression of the Th1-stimulating cytokine, IL-12 was observed in WC, when compared with weight gain in the absence of cycling (43). Besides, the percentages of abdominal and femoral subcutaneous adipose tissue pro-inflammatory CD3+CD8+ T cells did not change after weight loss (38).

### 4.3 Treg cell- obesity inflammation suppressor

Treg cell, known as a crucial immune-suppressive CD4+ T cell subset, is abundant in normal adipose tissue but decrease in obese mice. It was reported as a key suppressor of obesity-associated inflammation and metabolic abnormalities (46). In the T cell compartment, the greatest change in abundance occurs in Treg cells, which decrease with obesity and do not rebound with weight loss. Levels of adipose tissue Treg cells decline in proportion to the number of adipose tissue αβ T cells in obesity. Furthermore, during both weight loss and weight regain, their abundance remains lower than before the development of obesity (8). Based on these findings, we hypothesize that obesity may disrupt the anti-inflammation pathways of Treg cells, leading to WC.

### 4.4 CD7+ monocyte – weight cycling inhibitor

It was observed that the proportions of myeloid cells, including monocyte-macrophages and neutrophils, were significantly elevated in HFD and WC mice (47). In the classical monocyte subset, an increase in genes associated with lipid handling, activation/adhesion, and co-stimulation was not reversed with weight loss (19, 36). Functionally specialized monocyte subpopulation, CD7+ monocytes, exert distinct regulatory effects on weight metabolism. It was shown that compared to the individuals who were lean or obese, individuals post-dieting displayed increased CD7+ monocytes, which also had the effect of reducing the risk of weight regain (47). These cells, which accumulated metabolic memories through epigenetic adaptations, preferentially migrated to the subcutaneous white adipose tissue, where they facilitated beige fat thermogenesis in order to lose weight, through secreting fibrinogen-like protein 2 (FGL2) to activate the protein kinase A (PKA) signaling pathway (47). Mice transferred with CD7+ monocytes exhibited improved glucose tolerance, alleviated insulin resistance, and reduced tissue weights (47). CD7+ monocyte depletion induced weight regain rapidly, indicating it can not only treat but also prevent WC.

## 5 Potential intervention targeting immune memory

### 5.1 PAAu BPs - reprogramming macrophages

On the basis of the aforementioned theory, we can ameliorate WC through reducing the inflammation in adipose tissue. Gleeson et al (48) have found that the probability of weight regain was decreased through reprogramming the phenotype of macrophages from proinflammatory to anti-inflammatory. Emerging evidence suggested that immunotherapy strategy has been proposed for obesity treatment, and precise regulation of macrophage inflammation may provide a potential way to reduce obesity and WC (49). PAAu BPs, gold nanobipyramids engineered with adipose-targeting and apoptotic cell-mimicking functions, can activate macrophages to clear anceapoptotic camouflage of adipocytes (49). In the process of clearance, the macrophage turned from pro-inflammatory M1 to anti-inflammatory M2, significantly regulating the immune microenvironment of adipose tissues to prevent WC. Following inguinal injection with PAAu BPs, body weights of obese mice were effectively reduced by 24.4% and can be decreased by 33.3% when combined with photothermal lipolysis. Moreover, treatment with PAAu BPs under NIR laser irradiation was observed to noticeably inhibit weight regain during 15 days of treatment and another 15 days of weight regain monitoring period (49).

### 5.2 CD70-CD27 axis - reducing CD8+ T cell clonality

T cell memory is the basis of metabolic dysfunction resulting from WC. The CD27 receptor/CD70 ligand axis is one costimulatory interaction that promotes T cell memory formation (50). Low expression of CD27 was observed in NK cells, memory B cells, and quiescent T cells. The interaction between the co-stimulatory ligand CD70 (present on antigen-presenting cells) and CD27 (expressed on naïve CD4+ and CD8+ T cells) was critical for driving T cell proliferation, activation, and the establishment of T cell memory (51). It was found that blocking the CD70-CD27 axis decreased the number of memory T cells and restricted the ability of the T cell clone in adipose tissue following WC. CD70^-/-^ WC animals exhibited reduced CD8+ T cell clonality and improved metabolic consequences of WC for 27 weeks (52). Since CD70 plays a role in the development of obesity memory, inhibiting it may bring some benefits for individuals with WC.

## 6 Limitations and prospects

The focus of this review was mechanisms of immune memory-mediated WC and glucose and insulin disorders. We also recognize that this review also has several limitations. The information we have collected is mostly about animal experiments, while murine studies provide mechanistic insights into immune memory-induced WC, the mechanism of these findings in humans still remains to be established. Mechanistic studies on T cells-driven immune memory in WC remain to be more comprehensive in comparison to macrophages and more studies about the cellular specificity of CD8+ T cells on WC is needed. What’s more, the potential role of other immune cells is not extensively covered.

Promising research areas are as follows: clarifying antigens that cause immune memory to reduce the probability of WC; exploring the cellular specificity and more molecular targets of T cells in immune memory; conducting more studies to validate the safety and efficacy of interventions targeting immune memory-mediated WC; investigating the crosstalk between immune memory and metabolism of glucose and insulin to expand multi-target therapeutic strategies.

## 7 Conclusions

Immune memory serves as one of the key mechanisms linking WC, glucose and insulin metabolism. Initial metabolic stimuli induce pro-inflammatory memory in immune cells, which is activated during WC and then aggravates metabolic diseases through enhanced inflammation and damaged metabolic homeostasis. The concept of immune memory offers a novel perspective for explaining the refractory of weight regain and metabolic diseases.
