# Correction: The role of immune cells-mediated memory in weight cycling, glucose disorders and insulin resistance

**DOI:** 10.3389/fimmu.2025.1773368

**Published:** 2026-01-07

**Authors:** Yiding Chen, Dongqi Zhou, Lan Wang, Lisha Sun, Yun Yin, Guo Liu, Changyan Zi

**Affiliations:** 1 Qionglai Hospital of Traditional Chinese Medicine, Chengdu, Sichuan, China; 2 Hospital of Chengdu University of Traditional Chinese Medicine, Chengdu, China; 3 Sichuan Taikang Hospital, Tianfu New Area, Sichuan, China

**Keywords:** immune memory, weight cycling, macrophages, T-cells, insulin resistance

The funder “Chengdu Medical Research Project, 2025138” was erroneously omitted.

The original version of this article has been updated.

